# Implicit and Explicit Anti-Fat Bias among a Large Sample of Medical Doctors by BMI, Race/Ethnicity and Gender

**DOI:** 10.1371/journal.pone.0048448

**Published:** 2012-11-07

**Authors:** Janice A. Sabin, Maddalena Marini, Brian A. Nosek

**Affiliations:** 1 Department of Medical Education and Biomedical Informatics, University of Washington, Seattle, Washington, United States of America; 2 Department of Psychology, University of Virginia, Charlottesville, Virginia, United States of America; 3 Department of Communication and Economics, Università of Modena and Reggio Emilia, Reggio Emilia, Italy; 4 Department of Psychology, University of Virginia, Charlottesville, Virginia, United States of America; The University of Hong Kong, Hong Kong

## Abstract

Overweight patients report weight discrimination in health care settings and subsequent avoidance of routine preventive health care. The purpose of this study was to examine implicit and explicit attitudes about weight among a large group of medical doctors (MDs) to determine the pervasiveness of negative attitudes about weight among MDs. Test-takers voluntarily accessed a public Web site, known as *Project Implicit®,* and opted to complete the *Weight Implicit Association Test* (IAT) (N = 359,261). A sub-sample identified their highest level of education as MD (N = 2,284). Among the MDs, 55% were female, 78% reported their race as white, and 62% had a normal range BMI. This large sample of test-takers showed strong implicit anti-fat bias (Cohen’s *d = *1.0). MDs, on average, also showed strong implicit anti-fat bias (Cohen’s *d = *0.93). All test-takers and the MD sub-sample reported a strong preference for thin people rather than fat people or a strong explicit anti-fat bias. We conclude that strong implicit and explicit anti-fat bias is as pervasive among MDs as it is among the general public. An important area for future research is to investigate the association between providers’ implicit and explicit attitudes about weight, patient reports of weight discrimination in health care, and quality of care delivered to overweight patients.

## Introduction

In 2007–2008, the National Health and Nutrition Examination Survey estimated that 34.2% of US adults were overweight (BMI 25.0–29.9), 33.8% were obese (BMI≥30), and 5.7% were extremely obese (BMI≥40). (Centers for Disease Control and Prevention (CDC)) Prevalence of obesity among US children ages 2–19 is estimated at 17% with greater prevalence among minority children [1], [2]. In 33 US states, rates of obesity are ≥25%, and in nine states rates of obesity are ≥33% (CDC, Accessed March 3, 2011) In 2007–2008, the prevalence of obesity among white women was 33%, white men 31.9%, non-Hispanic black women 49.6%, non-Hispanic black men 37.3%, 45.1% among Hispanic women and 35.9% among Hispanic men. (Prevalence of Overweight, Obesity, and Extreme Obesity Among Adults: United States, Trends 1960–1962 Through 2007–2008. Centers for Disease Control and Prevention; June 2010. Accessed July 21, 2011).

Physicians agree that there is a necessity to treat obesity but many do not feel competent to do so [3]. In one study of primary care physicians, only 56% of physicians felt qualified to treat obesity [4]. In a systematic review of the literature, physicians’ perception of their competence to treat obesity in children and adolescents ranged from 5% to 33% [3]. A study of primary care physicians found that more than 50% viewed obese patients as awkward, unattractive and non-compliant [5]. More than one third of these physicians characterized obese patients as weak-willed, sloppy and lazy [5]. In another study, 45% of a sample of physicians agreed that they have a negative reaction to obese individuals [4]. A study of military family physicians found that physicians’ stereotypical attitudes of obese people as lazy increased 145% between 1998 and 2005, with younger physicians more likely to endorse this attitude [6].

The prevalence of weight discrimination among Americans has increased by 66% over the past 10 years [7]. Overweight patients report being treated disrespectfully by health professionals because of their weight [8]. One study found that 53% of overweight and obese women reported receiving inappropriate comments about weight from their doctors [9]. Obese patients who report perceptions of weight discrimination avoid seeking routine preventive care such as cancer screenings [10], [11].

In addition to self-reported beliefs, people also possess implicit or unconscious beliefs or biases that exist in memory but are often distinct from conscious values and beliefs. Implicit bias may predict discrimination behavior even among individuals who have no intention to discriminate [12]. In socially sensitive areas such as interracial attitudes and beliefs, implicit attitudes are a better predictor of discriminatory behavior than is self-report [13]. Implicit or unconscious negative attitudes about overweight people may be an under-recognized barrier to physicians engaging in weight management and appropriate communication about weight. Weight stigma in health care settings leads to poor quality of care for overweight patients [14]. Physicians’ implicit and explicit attitudes about weight remain understudied. One of the few published studies on implicit weight bias among health professionals found that they held negative implicit attitudes about weight [15]. Another study found that health professionals held implicit anti-fat attitudes associating “fat” with bad, lazy, stupid and worthless [16].

The purpose of this study was to examine implicit and explicit attitudes about weight among a large group of medical doctors (MDs) to determine the pervasiveness of negative attitudes about overweight among MDs. We examined and compared implicit and explicit attitudes about weight among a large convenience sample of the general population (N = 359,261), and a large sub-sample of MDs (N = 2,284) who chose to take the Weight Implicit Association Test (IAT) by accessing the *Project Implicit®* Web site (https://implicit.harvard.edu/). We disaggregated weight bias by MD gender, BMI, and race/ethnicity. We hypothesized that, similar to others in society, MDs would implicitly prefer thin people to fat people [17]. We explored whether MDs implicit attitudes favoring thin people rather than fat people would be similar to an expected explicit self-reported preference for thin people. The University of Virginia Institutional Review Board for the Social and Behavioral Sciences approved this research.

## Methods

### Sample and Procedures

Test-takers voluntarily, and without any recruitment from the researchers, accessed a public Web site, *Project Implicit* (https://implicit.harvard.edu), between May 2006 and October 2010 and chose to take the Weight Implicit Association Test. Participants accessed the *Project Implicit* site because of recommendations from others, a classroom assignment, media coverage, random web surfing, and many other mechanisms. As a volunteer and non-targeted sample, the sample is large and diverse; however, it should not be mistaken for a representative population sample. The size and diversity of the sample provides the opportunity to extend generalization of laboratory investigations that often use small, non-diverse samples. Test-takers were asked their age, gender, race, ethnicity, height/weight, country of residence, highest level of education and other characteristics**.** We identified medical doctors (MDs) through their self-reported highest level of education.

### Measures

#### The Implicit Association Test (IAT)

The IAT is a widely used measure of implicit social cognition that measures relative association strengths between two pairs of concepts [18]. For example, for measuring implicit attitudes about weight the concepts are *thin people*, *fat people*, *good words*, and *bad words* [19]. The Weight IAT was developed by researchers at Yale University, Department of Psychology, Rudd Center for Food Policy and Obesity and first reported in the literature in 2001 [15]. Since then it has been made widely available for research and demonstration purposes on the Project Implicit web site. In the Weight IAT, test takers are required to quickly categorize pictures of overweight and thin people and value laden words as they appear on a computer screen by pressing one of two computer keys. In one condition, participants categorize pictures of overweight people and “good” words with one key and pictures of thin people and “bad” words with the other key. In a second condition, the key assignments are reversed, pictures of thin people and “good” words are categorized with one key and pictures of overweight people and “bad” words are categorized with the other key. The difference in the average response time between the two conditions is an indicator of the relative association strength or bias toward one group rather than the other. For example, people that categorize good words with thin people and bad words with fat people faster than the other condition are said to have an implicit preference for thin rather than fat people [18], [19]. The IAT has become widely accepted as a measure of implicit social cognition because it captures evaluations that are related but distinct from self-report [20], achieves good reliability in comparison with other implicit measures [19], [21], is relatively robust with repeated measures for pre-post evaluation [19], [22], and has predictive validity across a variety of topics [13]. The IAT has been used in health disparities research with physicians to measure implicit attitudes about race. One study found that physicians hold implicit race bias, similar to others in society [23], and recent research is showing that these attitudes affect medical care [24], [25].

#### Explicit Measure

Test takers reported their attitudes about weight by endorsing one answer from the following list; 1. I strongly prefer thin people to fat people. 2. *I moderately prefer thin people to fat people. 3. I slightly prefer thin people to fat people. 4. I prefer thin people and fat people equally. 5. I slightly prefer fat people to thin people. 6. I moderately prefer fat people to thin people. 7. I strongly prefer fat people to thin people.*

#### Calculation of Body Mass Index (BMI)

BMI was calculated according the centers for Disease Control and Prevention (CDC) formula of dividing weight in pounds (lbs) by height in inches (in) squared and multiplying by a conversion factor of 703. (BMI = weight (lb)/[height (in)] 2×703). For adults, BMI is interpreted using standard weight status categories that are the same for all ages and for both men and women. The categories are as follows; <18.5 = Underweight, BMI 18.5–24.9 = Normal, BMI 25.0–29.9 = Overweight and BMI ≥30.0 = Obese. (Calculation of BMI 2011; http://www.cdc.gov/healthyweight/assessing/bmi/adult_bmi/, Accessed December 13, 2011).

### Analysis

We used descriptive statistics (means, standard deviations, and frequencies), to characterize the sample. For the explicit measure we coded the seven-point response scale to range from −3 to +3, with zero indicating no relative preference for fat people vs. thin people. For this study, an explicit measure mean that differs positively from zero indicated an explicit preference for thin people over fat people. We compared means for the implicit and explicit measures for the complete sample of test takers, for the MD sub-sample and for the MD sub-sample disaggregated by MD gender, BMI, and race/ethnicity.

The IAT effect is calculated as the standardized difference in mean response time on two key conditions of the IAT, known as the IAT D score [26]. The IAT was analyzed according to the improved scoring algorithm [27] with the following features: responses faster than 350 milliseconds were removed, responses slower than 10,000 milliseconds were removed, and errors were replaced with the mean of the correct responses in that response block plus a 600 millisecond penalty. In addition, IAT scores were disqualified for any of the following criteria suggestive of careless participation: (1) going too fast (<350 ms) on more than 10% of the total test trials, (2) making more than 30% erroneous responses across the critical blocks.

The IAT D score ranges from −2 to +2, with zero indicating no relative preference between thin people and fat people. Positive scores indicate an implicit preference for thin people, negative scores indicate an implicit preference for fat people. Because large samples result in virtually all effects being statistically significant, this report emphasizes reporting of effect size. Cohen’s *d*, a standardized effect size measure, was calculated for each of the implicit and explicit measures for each group. Cohen suggested the following interpretation of *d* guidelines; *d* of 0.20 = small effect, *d* of 0.5 = medium effect, and *d* of 0.80 = large effect. [28] Pearson’s correlation coefficient *(r)* was used to characterize the relationship between implicit and explicit measures.

**Table 1:** Characteristics of Weight IAT Test-taker Sample.

	N (%)	Gender (%)	Mean Age (SD)	% Reside in US	Mean BMI (SD)
**All Test Takers**	359,261		26 (10.7)	86	25 (5.9)
Male	83,348	27	26 (11.0)	84	25 (5.2)
Female	220,874	73	26 (10.7)	87	25 (6.1)
**All MDs**	2,284		34 (11.5)	78	24 (4.7)
Male	1.020	45	36 (10.3)	77	25 (5.1)
Female	1.285	55	33 (12.5)	79	24 (4.0)
**All Test Takers by BMI**		**% Female**			
Underweight	17,304 (6)	82	20 (6.6)	83	17 (1.0)
Normal	166,987 (58)	75	24 (9.1)	88	22 (1.7)
Overweight	59,370 (21)	62	28 (11.8)	89	27 (1.4)
Obese	44,439 (15)	74	32 (12.2)	92	36 (5.0)
**All MDs by BMI**					
Underweight	72 (3)	93	29 (8.8)	61	18 (1.0)
Normal	1,290 (62)	62	33 (10.6)	83	22 (1.7)
Overweight	507 (24)	35	37 (11.7)	80	27 (1.4)
Obese	221 (11)	53	40 (12.8)	86	35 (4.6)
**All Test Takers by Race/Ethnicity**					
White	231,807 (82)	72	26 (11.1)	86	25 (5.6)
African American	18,487 (7)	77	27 (10.9)	95	28 (7.0)
Asian	16,652 (6)	66	23 (7.7)	68	22 (4.3)
Hispanic	14,859 (5)	74	23 (7.9)	94	25 (5.9)
**All MDs by Race/Ethnicity**					
White	1,659 (78)	53	35 (11.9)	79	25 (4.8)
African American	104 (5)	68	34 (10.8)	87	26 (5.2)
Asian	319 (15)	60	30 (8.7)	78	23 (3.8)
Hispanic	39 (2)	47	32 (9.3)	77	26 (4.8)

BMI: Calculated using the CDC Formula: weight (lb)/[height (in)] 2×703.

## Results

Among the complete sample of test-takers (N = 359,261), 73% were female, 86% resided in the US, mean age was 26 years (SD = 10.7) and mean BMI was 25 (SD = 5.9). **Table 1** **.** Among all test-takers, 58% had a BMI in the normal range. Race/ethnicity of the complete sample was 82% white, 7% African American, 6% Asian, and 5% Hispanic. Among the sub-sample that identified their highest level of education as MD (medical doctor), 55% were female, 78% reside in the US, mean age was 34 years (SD = 11.5) and mean BMI was 24 (SD = 4.7). MDs reported their race/ethnicity as; 78% white, 5% African American, 15% Asian, and 2% Hispanic. Among MDs, 62% reported a BMI in the normal range versus 58% in the complete sample of test-takers. BMI distribution for the complete sample was as follows; 58% normal weight, 6% underweight, 21% overweight and 15% obese. BMI distribution for the MD sample was as follows; 62% normal weight, 3% underweight, 24% overweight and 11% obese.

### Implicit Measures

Results for large general population samples of Weight IAT test takers have been reported elsewhere [17]. These reports show that the general population holds strong implicit and explicit anti-fat bias. Similar to previous reports, this study’s large sample of test-takers showed strong implicit anti-fat bias (Cohen’s *d = *1.0). **Table 2** **.** MDs, on average, also showed strong implicit anti-fat bias (Cohen’s *d = *0.93). Implicit attitudes about weight among all test-takers were strong among both males and females. Among all female test takers and female MDs’ implicit anti-fat bias was significantly weaker than for males (*p<*.01, and *p<*.01). Although there were differences by gender, implicit anti-fat bias was strong among both male and female MDs (Cohen’s *d = *1.02 for males and Cohen’s *d = *0.86 for females). These differences by gender remained significant after adjustments for multiple comparisons. **Table 3** **.**

**Table 2:** Implicit and Explicit Attitude Measures for All Test Takers and All MDs (medical doctors) and by Gender.

IMPLICIT					EXPLICIT			
(Weight IAT)				Effect size	(Self-Report)			Effect size
All Test Takers	F	df	p	(a) ηp^2^	F	df	p	ηp^2^
**One-way ANOVA**	442,394	1, 304,220	<0.01	0.01	11099.137	1, 310333	<0.01	0.035
	**N**	**(b, c) Mean**	**SD**	**(d) Cohen’s ** ***d***	**N**	**Mean**	**SD**	**Cohen’s ** ***d***
**All Test Takers**	359,261	0.41	0.41	1.00	331,123	0.99	1.10	0.90
Male	83,348	0.45	0.42	1.07	85,520	1.32	1.15	1.15
Female	220,874	0.41	0.41	1.00	224,815	0.87	1.04	0.84
**All MDs**	**F**	**df**	**p**	**(a) ηp^2^**	**F**	**df**	**p**	**ηp^2^**
**One-way ANOVA**	21.219	1, 2276	<0.01	0.01	83.532	1, 2290	<0.01	0.35
	**N**	**(b, c) Mean**	**SD**	**(d) Cohen’s ** ***d***	**N**	**Mean**	**SD**	**Cohen’s ** ***d***
**All MDs**	2,284	0.40	0.43	0.93	2,297	1.36	1.08	1.24
Male	1,020	0.45	0.44	1.02	1,046	1.58	1.09	1.44
Female	1,258	0.37	0.43	0.86	1,246	1.17	1.04	1.13

a. ηp^2^ is the effect magnitude in a univariate regression with gender as the single predictor.

b. Implicit and explicit measures range from −2 to +2, with zero indicating no bias.

c. A positive mean indicates some degree of preference for “Thin” persons, a negative mean indicates some degree of preference for “Fat” persons.

d. Cohen’s *d* is a standardized effect size, comparing the means to M = 0 (no bias), interpreted as; *d* of 0.2 = small effect, *d* of 0.5 = medium effect, and *d* ≥0.8 = large effect.

**Table 3:** Pairwise Comparisons of Implicit and Explicit Weight Attitudes for All Test Takers and MDs (medical doctors) as a function of Gender, and for MDs as a function of BMI and Race/Ethnicity categories.

			IMPLICIT				EXPLICIT			
			(Weight IAT)				(Self-report)			
	Category	Comparison	MD (a)	SE	p*	CI	MD (a)	SE	p*	CI
**All**	**Gender**	Female vs. Male	0.04	0.00	**0.01**	**[0.03, 0.04]**	0.45	0.00	**0.01**	**[0.45, 0.46]**
**MD (b)**		Female vs. Male	0.08	0.02	**0.01**	**[0.05, 0.12]**	0.41	0.05	**0.01**	**[0.32, 0.49]**
**MD**	**BMI**	Underweight vs. Normal	0.07	0.05	0.90	[−0.06, 0.21]	0.04	0.12	1.00	[−0.28, 0.36]
		Underweight vs. Overweight	0.04	0.05	1.00	[−0.10, 0.18]	0.18	0.13	0.93	[−0.16, 0.52]
		Underweight vs. Obese	0.10	0.06	0.56	[−0.06, 0.10]	0.76	0.14	**0.00**	**[0.39, 1.13]**
		Normal vs. Overweight	0.04	0.02	0.63	[−0.02, 0.10]	0.14	0.05	**0.05**	**[0.00, 0.28]**
		Normal vs. Obese	0.17	0.03	**0.00**	**[0.09, 0.25]**	0.72	0.08	**0.00**	**[0.52, 0.92]**
		Overweight vs. Obese	0.14	0.03	**0.01**	**[0.05, 0.23]**	0.58	0.08	**0.00**	**[0.36, 0.80]**
**MD**	**Race/**	White vs. African American	0.07	0.04	0.54	[−0.04, 0.19]	0.39	0.11	**0.00**	**[0.11, 0.67]**
	**Ethnicity**	White vs. Asian	0.10	0.03	**0.01**	**[0.03, 0.17]**	0.05	0.07	1.00	[−0.13, 0.22]
		White vs. Hispanic	0.02	0.07	1.00	[−0.16, 0.21]	0.18	0.17	1.00	[−0.26, 0.62]
		African American vs. Asian	0.02	0.05	1.00	[−0.11, 0.15]	0.35	0.12	**0.02**	**[0.03, 0.66]**
		African American vs. Hispanic	0.05	0.08	1.00	[−0.16, 0.27]	0.57	0.20	**0.02**	**[0.06, 1.09]**
		Asian vs. Hispanic	0.08	0.07	1.00	[−0.12, 0.27]	0.23	0.18	1.00	[−0.24, 0.69]

* Adjustment for multiple comparisons: Bonferroni.

(a) Mean Difference.

(b) medical doctor.

For the MD sub-sample, implicit anti-fat bias was strong among underweight, normal weight and overweight medical doctors. **Table 4** **.** Among the 11% of MDs whose BMI classified them as obese, implicit anti-fat bias was moderate (Cohen’s *d = *0.60). After adjustments for multiple comparisons, variations in implicit bias by MD weight remained significantly different between normal weight vs. obese MDS (*p<*.01) and overweight and obese MDs (*p = *.01). This pattern of strong anti-fat bias among all weight groups except for obese individuals was similar among the complete sample of test-takers. (results not shown) Strong implicit anti-fat bias was found among white (Cohen’s *d = *1.0), and Hispanic (Cohen’s *d = *0.93) MDs. **Table 5** **.** After adjusting for multiple comparisons, there was a significant difference in implicit anti-fat bias between white and Asian MDs (*p* = .01). In the complete sample of test-takers all racial/ethnic groups showed strong implicit anti-fat bias. (not shown).

**Table 4:** Implicit and Explicit Attitude Measures for MDs (medical doctors) by BMI.

IMPLICIT					EXPLICIT			
(Weight IAT)				Effect size	(Self-Report)			Effect size
One-way ANOVA	F	df	p	(a) ηp^2^	F	df	p	ηp^2^
	10.556	3, 2086	<0.01	0.02	30.515	3, 2147	<0.01	0.04
**MD Weight (BMI)**	**N**	**(b, c) Mean**	**SD**	**(d) Cohen’s ** ***d***	**N**	**Mean**	**SD**	**Cohen’s ** ***d***
Underweight	72	0.36	0.40	0.90	77	1.52	1.06	1.43
Normal	1,290	0.44	0.42	1.05	1,320	1.48	1.02	1.45
Overweight	507	0.40	0.45	0.89	533	1.34	1.08	1.24
Obese	221	0.27	0.45	0.60	221	0.76	1.13	0.67

a. ηp^2^ is the effect magnitude in a univariate regression with BMI as the single predictor.

b. Implicit and explicit measures range from −2 to +2, with zero indicating no bias.

c. A positive mean indicates some degree of preference for “Thin” persons, a negative mean indicates some degree of preference for “Fat” persons.

d. Cohen’s *d* is a standardized effect size, comparing the means to M = 0 (no bias), interpreted as; *d* of 0.2 = small effect, *d* of 0.5 = medium effect, and *d* ≥0.8 = large effect.

**Table 5:** Implicit and Explicit Weight Attitude Measures for MDs (medical doctors) by Race and Ethnicity Category.

IMPLICIT					EXPLICIT			
(Weight IAT)				Effect size	(Self-Report)			Effect size
One-way ANOVA	F	df	p	(a) ηp^2^	F	df	p	ηp^2^
	5.182	3, 2117	<0.01	0.01	5.009	3, 2136	<0.01	0.01
**MD Race/Ethnicity**	**N**	**(b, c) Mean**	**SD**	**(d) Cohen’s ** ***d***	**N**	**Mean**	**SD**	**Cohen’s ** ***d***
White	1,659	0.43	0.43	1.00	1,666	1.39	1.05	1.23
African American	104	0.35	0.49	0.71	107	1.00	1.28	0.78
Asian	319	0.33	0.43	0.76	325	1.34	1.07	1.25
Hispanic	39	0.40	0.43	0.93	42	1.57	1.15	1.37

a. ηp^2^ is the effect magnitude in a univariate regression with race/ethnicity as the single predictor.

b. Implicit and explicit measures range from −2 to +2, with zero indicating no bias.

c. A positive mean indicates some degree of preference for “Thin” persons, a negative mean indicates some degree of preference for “Fat” persons.

d. Cohen’s *d* is a standardized effect size, comparing the means to M = 0 (no bias), interpreted as; *d* of 0.2 = small effect, *d* of 0.5 = medium effect, and *d* ≥0.8 = large effect.

### Explicit Measures

All test-takers reported a strong explicit preference for thin people over fat people on average (Cohen’s *d = *0.90). There was variation in self-reported anti-fat bias among all test takers by gender, however, the variation was within the range of strong bias (Cohen’s *d = *1.15 for males and Cohen’s *d = *0.84 for females). We found strong self-reported anti-fat attitudes among MDs by gender (Cohen’s *d = *1.44 for males and Cohen’s *d = *1.13 for females). These differences by gender for all tests takers and for MDs remained significant after adjustments were made for multiple comparisons. For MDs who were underweight, normal weight and overweight we found strong explicit anti-fat bias. Among the sub-sample of MDs who were obese, we found moderate explicit anti-fat attitudes (MD sample Cohen’s *d = *0.67*),* similar to all obese test-takers (not shown). After adjusting for multiple comparisons, we found a significant difference in strength of self-reported anti-fat attitudes between underweight vs. obese MDs (*p<*.001), normal vs. overweight MDs (*p = *.05), normal vs. obese MDs (*p<*.001), and overweight vs. obese MDs (*p<*.001). We found a significant difference in self-reported weight bias among white and African American MDs (*p<*.001), African American and Asian MDs (*p = *.02), and African American and Hispanic MDs (*p = *.02) although all groups showed strong anti-fat bias.

We found a statistically significant, but modest relationship, between implicit and explicit measures for the complete sample of test-takers (*r = *.20, *p<*.01), and for the MD sub-sample (*r = *.23, *p<*.01). **Table 6** **.** This pattern was found among MDs by MD gender, BMI, and for white and Asian MDs. There was no significant relationship between implicit and explicit attitudes about weight among African American MDs (*r = *.14, *p = *.17) and Hispanic MDs (*r = *.01, *p = *.95), though those groups were much smaller samples than the others.

**Table 6:** Inter-correlation Between Implicit and Explicit Weight Attitude Measures.

Implicit/Explicit Attitude Correlation	N	*r* (b)	p
**All Test Takers**	297,161	0.20	<0.01
**All MDs**	2,166	0.23	<0.01
**MD Gender**			
Male	965	0.24	<0.01
Female	1,196	0.19	<0.01
**MD BMI**			
Underweight	71	0.13	<0.01
Normal	1,255	0.23	<0.01
Overweight	494	0.15	<0.01
Obese	211	0.23	<0.01
**MD Race/ethnicity**			
White	1,577	0.22	<0.01
African American	99	0.14	0.17
Asian	307	0.24	<0.01
Hispanic	37	0.01	0.95

a. N represents total test takers for whom we have both implicit and explicit measures.

b. Pearson’s correlation *(r).*

## Discussion

This study is the first to show strong implicit and explicit weight bias among a very large sample of MDs by MD’s BMI, race/ethnicity and gender. Our study extends prior knowledge of physicians’ implicit and explicit attitudes about weight measured by the IAT, by comparing a very large sample of MDs with a very large sample from the general population and by MD’s personal characteristics. The results for MDs are similar to results from large samples of the general public who voluntarily take the Weight Implicit Association Test at the *Project Implicit®* web site [29]. We found that MDs’ implicit and explicit attitudes about weight follow the same general pattern seen in the very large public samples that hold strong implicit and explicit anti-fat bias. We conclude that implicit and explicit anti-fat bias is as pervasive among MDs as it is among most people in society. In socially sensitive areas such as race, sexuality, disability, and age, implicit attitudes are often stronger than self-reported attitudes [30]. However, for weight bias, both implicit and explicit anti-fat attitudes are very strong, with self-reported attitudes slightly stronger. Strong explicit attitudes suggest that individuals, including medical doctors, may feel that it is socially acceptable to express negative attitudes about overweight people.

Our study found strong implicit and explicit weight bias among male and female MDs, similar to all test takers in the sample, with females showing less implicit bias. Although weight bias was strong among all MD test takers, we found significant variations in strength of implicit and explicit weight bias by MD BMI and race/ethnicity. We do not yet know how MDs implicit and explicit anti-fat attitudes affect interpersonal clinical behavior. Nor do we know whether implicit weight bias is related to how overweight patients experience health care interactions. However, parallel evidence with implicit racial biases suggest that these are important areas to investigate [31].

An important limitation of this study is that the sample is not a random, representative sample of MDs. Thus, the sample means and distributions cannot be considered parameter estimates of MDs in general. It is possible that due to selection effects, the presence of implicit weight bias among MDs is either underestimated or overestimated. We do not know who chose to voluntarily self-assess their implicit weight bias by taking the Weight IAT, but we expect that the sample does not include the most biased individuals in society. However, because the analysis included more than 2,200 MDs, the present findings show that implicit and explicit weight bias among medical doctors is widespread.

Although we are relying on self-report of MD status, which is imprecise, some research shows that for sensitive topics, Web test takers are more likely to be honest than face-to-face or telephone survey respondents [32], [33], [34], [35]. Data collected from *Project Implicit®* has been studied intensively for several years and the validity of results is comparable to that of similar data collected in experimental laboratory conditions [26], [36]. Several studies that compare information provided by Web responders with information from traditional methods found no difference in validity of the data collected [37], [38]. While there is a slightly higher probability of test takers providing misinformation than in more controlled conditions [37], this issue is not a factor in the interpretation of very large data sets [19], [39]. The quality of data collected on the Web is often better than the quality of data gathered using other methods because the human error rate in the data collection process is reduced [32], [35].

Despite its limitations, this study is the first research to measure implicit and explicit attitudes about weight among a very large group of MDs to demonstrate just how pervasive these attitudes are among MDs and compare them to the general population. Strong negative implicit and explicit attitudes about weight among MDs may contribute to less-than-ideal clinical interactions and subsequent medical avoidance among overweight patients. Exploring the effects of physicians’ implicit and explicit weight bias on quality of care of overweight patients is an important area for future study.

Physicians express great interest in additional training in obesity management [40]. In one study, one fourth of physicians (pediatricians and family medicine physicians) report that they are “not at all” or only “slightly competent” when addressing obesity and that treating obesity is “very frustrating” [4]. Medical residents do not feel they are competent in weight management [41]. In one study, only 23.6% of clinicians reported receiving training in good obesity practices in medical school and 30.9% in residency training [42]. Those who reported receiving training in good obesity practices were more likely to discuss diet and exercise with obese patients.

Education programs for physicians and other health professionals, which often focus on causes and treatment of obesity, should also aim to increase clinicians’ self-awareness about implicit and explicit personal attitudes about weight and identify how these attitudes may affect care delivered to overweight patients. Some evidence suggests that explicit attitudes are more related to deliberate and verbal behavior and implicit attitudes are more related to spontaneous and non-verbal behavior [43]. Educational interventions for providers that focus on weight management should emphasize improving clinicians’ verbal and non-verbal communication skills in the prevention and management of overweight. An important area for future research is to investigate the links among clinicians’ implicit and explicit attitudes about weight, patient reports of weight discrimination in health care, and quality of care delivered to overweight patients.
